# Real Time Monitoring of Children, and Adults with Mental Disabilities Using a Low-Cost Non-Invasive Electronic Device

**DOI:** 10.3390/mi8100292

**Published:** 2017-09-28

**Authors:** Carlos Polanco, Ignacio Islas Vazquez, Adrian Martinez-Rivas, Miguel Arias-Estrada, Thomas Buhse, Juan J. Calva, Carlos Aguilar Salinas, Claudia Pimentel Hernández, Vladimir N. Uversky

**Affiliations:** 1 Department of Mathematics, Faculty of Sciences, Universidad Nacional Autónoma de Mexico, Mexico City 04510, Mexico; 2 Escuela Superior de Ingeniería Mećanica y Eléctrica, Instituto Politécnico Nacional, Mexico City 07738, Mexico; nachoislas@yahoo.com; 3 Centro de Investigación en Computación, Instituto Politécnico Nacional, Mexico City 07738, Mexico; nanobiomex@hotmail.com; 4 Department of Computer Science, Instituto Nacional de Astrofísica, Óptica y Electrónica, Puebla 72840, Mexico; ariasmo@inaoep.mx; 5 Centro de Investigaciones Químicas, Universidad Autónoma del Estado de Morelos, Morelos 62209, Mexico; buhse@uaem.mx; 6 Department of Infectious Diseases, Instituto Nacional de Ciencias Médicas y Nutrición Salvador Zubirán, Mexico City 14080, Mexico; juanjcalva@gmail.com; 7 Unidad de Investigación de Enfermedades Metabólicas, Instituto Nacional de Ciencias Médicas y Nutrición Salvador Zubirán/Instituto Tecnológico y de Estudios Superiores de Monterrey TecSalud, Mexico City 14080, Mexico; caguilarsalinas@yahoo.com; 8 Unidad de Apoyo a la Investigación Clínica, Instituto Nacional de Pediatría, Mexico City 04530, Mexico; clauspim@hotmail.com; 9 Department of Molecular Medicine and USF Health Byrd Alzheimer’s Research Institute, Morsani College of Medicine, University of South Florida, Tampa, FL 33647, USA; vuversky@health.usf.edu; 10 Institute for Biological Instrumentation, Russian Academy of Sciences, 142290 Moscow Region, Russia

**Keywords:** electronic devices, real time monitoring, mental disabilities, children, Arduino^®^ platform

## Abstract

There are a growing number of small children—as well as adults—with mental disabilities (including elderly citizens with Alzheimer’s disease or other forms of age-related dementia) that are getting lost in rural and urban areas for various reasons. Establishing their location within the first 72 h is crucial because lost people are exposed to all kinds of adverse conditions and in the case of the elderly, this is further aggravated if prescribed medication is needed. Herein we describe a non-invasive, low-cost electronic device that operates constantly, keeping track of time, the geographical location and the identification of the subject using it. The prototype was made using commercial low-cost electronic components. This electronic device shows high connectivity in open and closed areas and identifies the geographical location of a lost subject. We freely provide the software and technical diagrams of the prototypes.

## 1. Introduction

The loss of children and elderly people with mental disability (including elderly citizens with Alzheimer’s disease or other forms of age-related dementia) has a high emotional and financial impact on their relatives and communities. In Mexico City, the annual incidence is 1500 individuals [1], of whom only 50% are eventually located [2]. Furthermore, according to the World Health Organization (WHO), 47.5 million people suffer from dementia worldwide, and 7.7 million new cases are recorded annually. With the aging of our societies, the number of elderly people with mental disabilities grows [2,3,4]. In developed countries, social security programs protect both vulnerable groups (children and elderly people with mental disability) with day nurseries, asylums and hospices, however this is not the case in developing countries, where the health care facilities to provide care for these people are almost non-existent, and children and adults with disabilities are often cared for by indirect relatives during working hours. This paper describes a very low cost electronic non-invasive device (patent pending) that can be provided to both groups at risk, which works 24 h a day, every day, in real time. This device, in the case of an event, will activate and transmit the time-space location of the subject and its identification. The device does not use a Global Positioning System (GPS) tracker, but utilizes the location of an independent receiver module that can be installed in shopping malls, electricity towers, bus stations, and traffic lights among other suitable sites. The battery life of this electronic device is five days, which guarantees a response within the first 72 h, which is critical for the location of the lost subject. The electronic schemes and the programs written in Arduino^®^ language are provided in the Supplementary Materials.

## 2. Material and Methods

**Figure 1:**
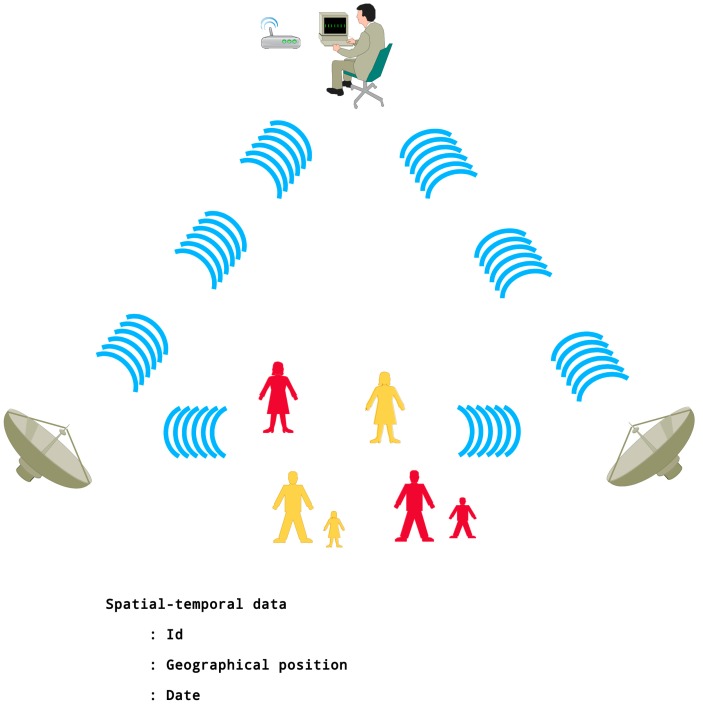
Monitoring collection options.

**Figure 2:**
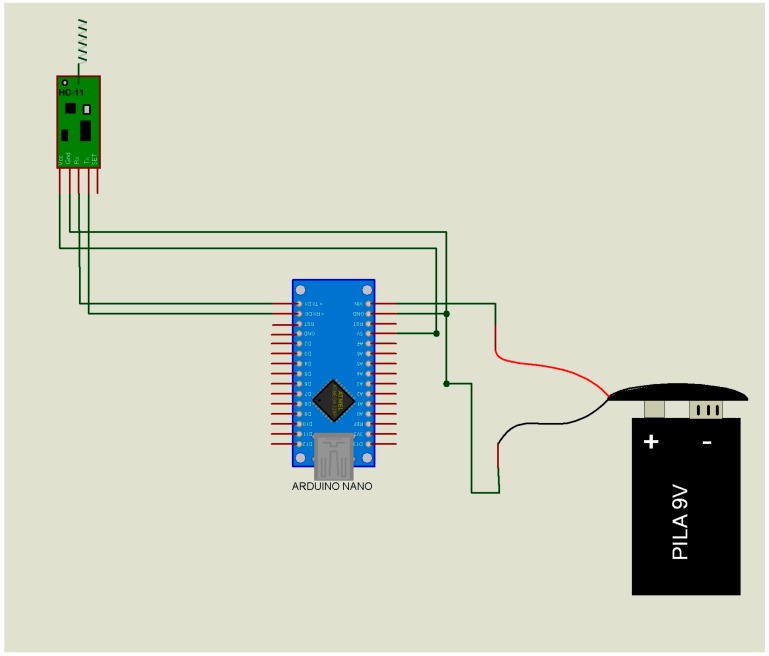
Schematic diagram of the data receiver/transmitter module or “slave”. Receiver mode: the missing people file from master modules. Transmitter mode: the subject’s identity data is sent to the master module only in case the subject is searched (Table 1 (A,B)). Figure taken and adapted from Polanco et al. [5].

**Figure 3:**
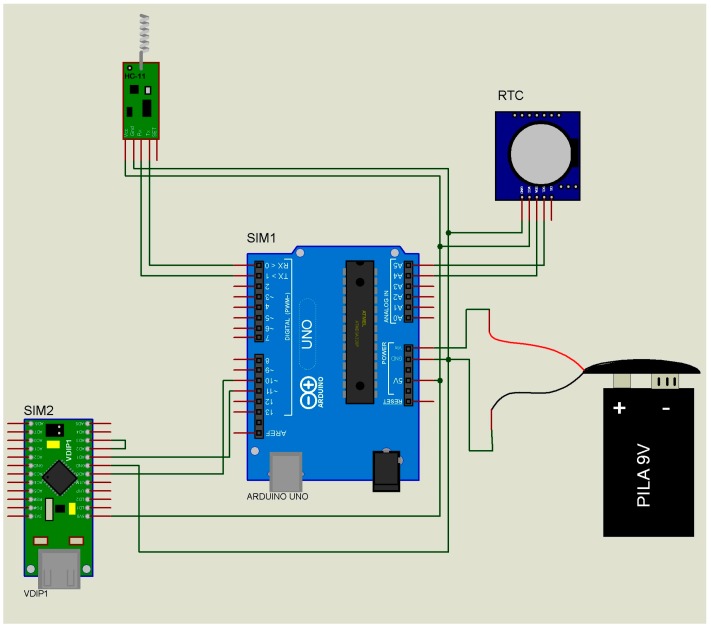
Schematic diagram of the data receiver/transmitter module or “master.” Receiver mode: missing people file from the supervisor module (Table 2 (A)). Transmitter mode: people found by the slave modules sent to the supervisor module and the missing people file sent to the slave modules (Table 2 (B,C)). Figure taken and adapted from Polanco et al. [5].

**Figure 4:**
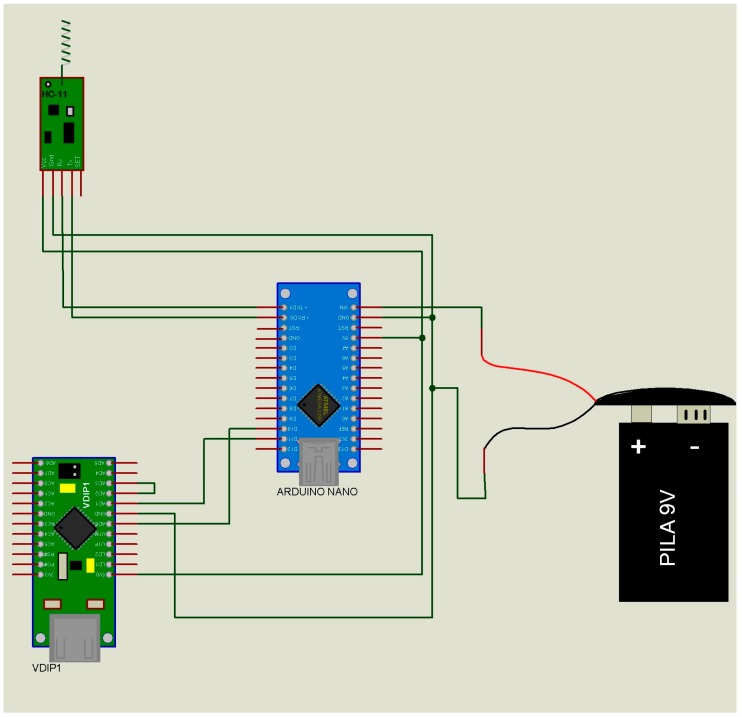
Schematic diagram of the data receiver/transmitter module or “supervisor”. Receiver mode: file of people found by the master modules (Table 4 (A)). Transmitter mode: missing people file sent to the master modules (Table 4 (B)). Figure taken and adapted from Polanco et al. [5].

The prototype (Figure 1) is comprised of three modules: a data transmitter (Figure 2), a data receiver (Figure 3), and a data supervisor (Figure 4). It was inspired by the previous work of Polanco et al. [5].

1. The data transmitter module or “slave” (Figure 2) has only one function, namely to send the ID of the subject to the data receiver module or “master” (Table 1 (A,B)). When the “master” receives this ID, it emits a confirmation. If the ID does not match, there will be no message confirmation. This passive way of communicating will substantially reduce the power consumption of the “slave” device of the end users (children and adults).
2. The data receiver module or “master” (Figure 3) performs three functions: (i) It receives the list of the IDs to be located from the “supervisor”; (ii) It sends the ID searched to all the “slaves” (Table 2 (A)); (iii) in the case of successfully identifying a “slave” (Table 2 (B,C)), it sends to the data “supervisor” module the “slave” ID, the “master” ID, and the recording date and time (year, month, day, hour, minute) (Table 3). This is the device that has to be installed at fixed sites and whose geographical address is transmitted to the “slaves”.
3. The data “supervisor” module (Figure 4) has three functions: (i) It updates the list of IDs that have to be located (Table 4 (A)); (ii) It sends this list of IDs to the “masters” (Table 2 (C)); and (iii) It receives the list of the located IDs from the “masters” (Table 4 (B)). This device updates the list of missing individuals from “masters”, and has the list of individuals located by the “slaves.”

***On-Site testing***. Two “masters,” two “slaves,” and one “supervisor” module were tested outdoors and indoors at peak pedestrian and traffic hours.

***Indoor areas only***. Monitoring was performed in different areas inside the building site, delimited by fixed walls at peak hours of use (Figure 1). The “masters” (Figure 3) were fixed to different walls inside the building and the “slaves” (Figure 2) were located on different floors. The “supervisor” (Figure 4) was also placed inside the building site.

***Indoor/outdoor areas***. Monitoring was performed in different areas inside the building site and at the parking lot (Figure 1). The “slaves” (Figure 2) were placed outside (parking lot), and the “masters” (Figure 3) were positioned inside the building. The “supervisor” (Figure 4) was placed outside the building site.

***Outdoor areas only***. Monitoring was performed in the vicinity of the building site (Figure 1). The two “slaves” (Figure 2) were placed at the sides of the building and the “masters” (Figure 3) were installed on the top of the building (Figure 1) 20 m above the “slaves” (Figure 2). The “supervisor (Figure 4) was placed inside the building site.

***Data.*** The location records (Table 3) were generated by the “slaves” and transmitted to the “masters,” where the corresponding masters’ IDs were added, and the information was finally sent to the “supervisor”.

***Modes.*** Data transmission between the three modules was carried out in two different ways:

***Active mode***. When a module responds to another module. This is when a “slave” confirms a “master” and when a “master” confirms a “supervisor”.

***Passive mode****,* when a module does not respond to another module, that is when there is no confirmation from the “slave” to the “master” module. This mode saves energy. 

The transmission between the different modules, was executed with a HC-11 434MHz Wireless Serial Port (http://wiki.seeedstudio.com/images/a/a8/HC11_User_Manual.pdf), baud rate 9600 equivalent to range 200 m, and frequency band 434 M. This device does not use a GPS tracker, instead using the physical address of master modules—this avoids including a GPS tracker, the cost of which is high.

## 3. Results

There was no important loss of connectivity in any of the three modules “slave,” “master” or “supervisor” (Table 2, Table 3 and Table 4), in any of the three modes: indoor, indoor/outdoor, and outdoor areas. The test for battery lifetime in the “slaves” was 5 days in passive mode and 3 days in active mode. The test for battery connectivity in the “masters” did not show any connectivity disruption. Replacing the coordinates of the GPS tracker in the “slave” module by the code of the physical location of the ‘master,’ reduced from 30 to 2 bytes the amount of data transferred. This increased the data transmission gain and expanded the range of the signal compared to our previous work Polanco et al. [5], where it was used for wireless transmission by radio-frequency an HC-11 434 MHz Wireless Serial Port (http://wiki.seeedstudio.com/images/a/a8/HC11_User_Manual.pdf), baud rate 9600 equivalent to range 200 m, and frequency band 434 M.

## 4. Discussion

***Test plan focus***. The tables describing the data for the test is the information transmitted between the different modules. This electronic application derives from a previous work from this group Polanco et al. [5], where the transmission by wireless radio-frequency was extensively verified through an HC-11 434 MHz Wireless Serial Port (http://wiki.seeedstudio.com/images/a/a8/HC11_User_Manual.pdf), baud rate 9600 equivalent to range 200 m, and frequency band 434 M. In that previous work, it was discussed and proved the efficiency and integrity of this type of transmission. In this improved version, the number of fields transferred between modules represents a fifth of the data transferred in the previous work. This is because the parameters of geolocation given by the GPS tracker were changed by the “master” module location so only 2 bytes of information were transferred instead of 30 bytes.

***Final costs***. The “slave,” “master,” and “supervisor” prototypes were built with an Arduino^®^ platform. Their costs were $10 USD, $20 USD, and $15 USD respectively. An estimate of the serial cost by unit would be $5 USD, $10 USD and $7.5 USD respectively if is implemented an application specific integrated circuit (ASIC). The information collected by the “supervisor” allows identification of the location of a subject within an area of 200 m. The duration of the battery charge would be 5 days. Another alternative would be to use solar energy for the “slave” modules. Further improvement could be reached by replacing the batteries of the “slave” modules by piezoelectric components, or solar membranes (that would stick to the clothes). This improvement allows recharging of the batteries and would extend the batteries more than five days, but the cost would increase.

***Population Benefits***. With an ever-increasing population in underdeveloped countries, they are facing the aging of their population. Therefore, it can be inferred that vulnerable groups in these nations will increase substantially in the next years. This will imply a double burden for these nations, where family members will have to pay for the medical care and surveillance of the vulnerable groups. Currently, there are several prototypes, that monitor one or several biometric data and/or carry out the tracking of people [6,7,8,9,10] via GPS trackers. However, the use of a device among large populations will necessarily require a very low cost, or to be offered free of charge. In addition, such a device will have to be harmless to the user. The prototype described here complies with both requirements.

***Mental disabilities in developing countries***. Mental disorders are a growing cause of premature disability worldwide. However, the trend is more pronounced in developing countries. The growth of life expectancy and the interaction with chronic non-transmissible disorders contributes to an increase in the number of cases with depression, dementia, or anxiety. The infectious disease-driven health care systems of developing countries are not prepared to face the complex challenges that represent the treatment of mental disorders [11,12,13].

***Time spent searching***. There are no strong records indicating the maximum time spent to find a lost person. However, it is clear that a “safe” time depends on their medical condition, the medical treatments required on a daily basis, as well as physical and mental impairments. If it is known that a person can walk on average one meter in three seconds, then in just one hour the subject can be found anywhere within an area of one kilometer in diameter, which is why the search has to start as soon as possible.

***Better Clinical Decision-Making***. The use of these devices in hospitals for hospitalized or ambulatory out-patients or as well as for children shelters would improve their monitoring, as it makes possible to know their whereabouts. This means that the receiver “masters” will have to be located in specific areas of the facilities. For instance, in Mexico there are high-referral centers with a relatively short capacity to hospitalize patients; not uncommonly, due to this sort of constrains individuals in critical clinical circumstances need to be sent to contiguous health care facilities and frequently their final destination is unknown, leading to unfavorable outcomes. Often, ambulatory patients fail to attend a scheduled appointment in out-patient clinics because they decide instead to go to emergency services with acute-onset signs and/or symptoms. In these circumstances an efficient, timely and accessible surveillance system is highly needed.

***Satisfaction/Acceptability***. The low cost and specifications of electronics involved will allow improvements in design to make it attractive and accepted in schools or hospitals, where the device will give a warning in the case where a subject tries to leave. This will require the installation of alarms in the receiver “masters,” located in places near the exits.

***Costs involved.*** The design of this prototype is formed by three modules: slaves, masters, and supervisor, its general design considers the minimization of hardware/software involved in order to minimize its cost, in this first stage, but also to ensure the fulfilment of the objective. There are better transceivers than transceiver HC-11, including shields enabled with all wireless communication protocols, however, the communication protocol to baud rate 9600 that offers the transceiver HC-11 is the minimum cost that allows us to cover the objectives of communication between each module.

***Future Trend for Electronic Devices in Health Care***. Bio-nano-robots, currently in full experimental development, will be capable of decision-making, movement, and action. These three qualities should be used to transform the device presented here into a device that also registers the biometric data of children and adults with mental disabilities, besides providing their geographical location in real time.

***Possible Applications in Translational Medicine***. During the last decade, great progress has been achieved in the field of portable devices design (wearable) and their systems, the research into which focuses on clinical applications [14] such as the geo-location of people with dementia or mental disabilities [15]. These devices have been widely studied in the United States, Canada, and China, their cost ranges from 16 to 1000 USD (see Supplementary Materials). In all cases, the tracking devices are potentially useful for providing the geo-location of patients and thus reducing institutional costs [15]. It is convenient to make sure these devices are practical and easy to use and that their cost, appearance, and reliability encourage their usage [16]. Current epidemiology surveillance systems based on a minimal number of subjects confirmed by lab tests face the fact of over-saturation due to a substantial increase in the transportation of large numbers of passengers [17,18,19]. With the use of the proposed device, the surveillance systems could be benefited by monitoring frequent travelers, since it could follow their geographical location [17,20,21] in real time, and thereby help preventing the spread of potential epidemic diseases.

***Automatic Alert System: An Improvement***. Besides the aforementioned improvements in energy supply for the device, it can also be adapted to current camera surveillance systems on city streets, as it can be easily interconnected to such systems. Its electronics, programming, and cost make it easy to adjust to any equipment used. It can also be implemented in automated tracker surveillance systems [18,22,23,24,25,26] to obtain the whereabouts of subjects.

***Millimeter-Scale Computers***. Electronics miniaturization continues to be the driving force of electronics and computer industries. Although claims about the end of Moore’s law since the late 90’s, progress has been steady, providing smaller and smaller components. Microprocessor complexity and computational density is a main motivation, but the side effect is that smaller transistors require more energy to operate, and low consumption devices have been pushing the technology in the mobile era. Wearable devices benefit from the technology progress since it is possible to integrate complex processors in millimetric scale systems. State of the art research in smart sensors is proving this [27]. On the other hand, the current interest in wearables have opened a wide possibility to design, miniaturize and create low cost devices. The monitor device in this work is around an Arduino® low cost that could be potentially reduced in size and power composition further. A whole line of alternatives ranges from Atmel microsystems [28], (the processor of the Arduino®, but also Microchip [29], NXP (focusing in a new line of wearable designed ICs, (NXP, 2017) [30], Intel (Intel Curie devices for wearables [31], and wearable/IoT IPs developed by Synopsys (https://www.synopsys.com/designware-ip/ip-market-segments/internet-of-things/wearable-fitness-health.html). Nevertheless, all alternatives still require power consumption and the logistics to maintain the discipline of charging batteries periodically. We can consider that miniaturization and low cost should go in hand with some way to guarantee a personal monitor with longer life and less dependency on the user or his/her family. An alternative is the use of supercapacitor or miniature rechargeable batteries [32] that could charge using the user’s motion. Key places to explore charging are hand/arm motion for bracelets and feet/walking energy generation in foot/shoe devices. The former is probably better in the short term, but the latter could be of interest since shoes are personal and most of the time are worn on the street. Walking motion with a piezoelectric battery charge has the most energy generation potential. If the cost is low enough, a patient could have redundant devices (different shoes, bracelet) so the risk of not having a functional or discharged sensor during an eventuality is minimized. Finally, the monitoring infrastructure should consider having a continuous sensor monitor status “heartbeat” to monitor the correct function of all deployed devices, and advice to the families if any of them is not working properly.

***Nanobots: The Next Frontier Approach***. Advances in electronics, nanotechnology and material science have permitted the implementation of some devices and systems related to nanorobot (nanobots), as they are thought to perform several medical functions such as killing cancer cells or transmitting information abour the interior of the body [33,34]. One practical example is the wireless capsule endoscope (WCE) that takes images of the internal gastrointestinal (GI) track [32], it has a small wireless data transmission system that could also be utilized to localize subjects. However, we are using carbon based nanomaterials such as graphene [35], because it is a low-cost material with high carrier mobility, high elasticity, to elaborate nanodevices, applied to biomedicine [36]. We are interested in elaborating, by micro-nanofabrication techniques, graphene antennas to succeed in having a wider broadband [37,38] and to be able to use graphene in wearable devices [39] or as an antenna patch [40], which would represent an improvement in the proposed device, for the real time monitoring of children and people.

***Biomarkers: Possible Uses***. Recent years witnessed increased interest in using disease biomarkers (i.e., specific proteins, or their fragments, or DNA/RNA, or small molecules generated by abnormal cells) as useful ways for better understanding disease biology and management [41]. Besides being indicators of specific biological states or pathological conditions, biomarkers provide useful information on the molecular mechanisms underlying initiation and development of disease as well serve as molecular signatures and specific time stamps of the physiological state of a disease, being therefore very important for both detection and staging of a malady [41,42,43,44]. Information on various biomarkers can be fed to the device described in this work.

***Tracker surveillance system***. The loss of a relative due to her/his mental disability, or of a child, is a major family problem, even if her/his rapid geo-location happens, the psychological damage to the family is permanent. The damage is major if the disappearance occurs for weeks or months, since she/he will be deprived of her/his medicines, or exposed to multiple threats. The design of this noninvasive device is of very low cost, so that it could be provided free of charge through clinics and shelters to a vulnerable population. The tracker surveillance system must be under custody of the federal government, since the system uses private data.

***Geo-location***. The geographical location of the “slave” module and the “master” module is the same. The master will identify the “slave” within a range of 200 m, if the carrier of the “slave” goes through the range of detection of two or more “masters,” the “supervisor” module will receive multiple time/space locations—these locations will allow the tracing of a “route” in time to determine the location of the carrier of the “slave.” The number of masters installed will improve the final location of the carrier.

***Ethical issue***. The final design of the device was as a noninvasive bracelet that does not limit the movement of the carrier. It will be activated only in the case of a carrier getting lost. The system will only locate the subject when the family issues a lost alert, preventing the use of the device as a pager.

## 5. Conclusions

This prototype proved to be a very low-cost device capable of locating vulnerable groups: children, and adults with mental disabilities, within the first 72 h since disappearance. Its final cost makes a convenient product to be offered free of charge to both vulnerable groups through the Health system, or can be adopted by associations responsible to those groups. The replacement of the GPS tracker by the physical location of the devices receiving the identity of the lost subject reduced the cost of the prototype, and increases the possibility of massive use.

## Figures and Tables

**Table 1:** Monitoring data frame. (**A**) Master 1. Slave A; (**B**) Master 2. Slave B.

Start	Master ID	Slave ID	DD	MO	YY	HH	MM	DF	End
(**A**)									
@	1	A	29	06	16	22	19	!	&
@	1	A	29	06	16	22	20	!	&
@	1	A	29	06	16	22	21	!	&
@	1	A	29	06	16	22	22	!	&
@	1	A	29	06	16	22	23	!	&
@	1	A	29	06	16	22	24	!	&
@	1	A	29	06	16	22	25	!	&
@	1	A	29	06	16	22	26	!	&
(**B**)									
@	2	B	29	06	16	22	19	!	&
@	2	B	29	06	16	22	20	!	&
@	2	B	29	06	16	22	21	!	&
@	2	B	29	06	16	22	22	!	&
@	2	B	29	06	16	22	23	!	&
@	2	B	29	06	16	22	24	!	&
@	2	B	29	06	16	22	25	!	&
@	2	B	29	06	16	22	26	!	&

Data collected by the data transmitter breadboard. **Start**: “@” starts recording information. **Master ID**: Device identification “1, 2”. **Slave ID**: Device identification “A, B”. **DD**: Recording day. **MO**: Recording month. **YY**: Recording year. **HH**: Recording hour. **MM**: Recording minutes. **DF**: “!” dummy field. **End**: “&” end of the recording.

**Table 2:** Monitoring data frame. (**A**) Master 1. Missing people; (**B**) Master 1. Found people; (**C**) Master 2. Found people.

Start	ID Master	ID Slave	DD	MO	YY	HH	MM	DF	End
(**A**)									
@		A						!	&
(**B**)									
@	1	A	29	06	16	22	19	!	&
@	1	A	29	06	16	22	20	!	&
@	1	A	29	06	16	22	21	!	&
@	1	A	29	06	16	22	22	!	&
@	1	A	29	06	16	22	23	!	&
@	1	A	29	06	16	22	24	!	&
@	1	A	29	06	16	22	25	!	&
@	1	A	29	06	16	22	26	!	&
(**C**)									
No data									

Data collected by the data transmitter breadboard. **Start**: “@” starts recording information **ID master**: Device identification “1, 2”. **ID slave**: Device identification “A, B”. **DD**: Recording day. **MO**: Recording month. **YY**: Recording year. **HH**: Recording hour. **MM**: Recording minutes. **DF**: “!” dummy field. **End**: “&” end of the recording.

**Table 3:** Monitoring.

Field	Symbol	Description
Identificator slave	ID slave	Alphabetic code that identifies the “slave” data transmitter module. This code has the personal information and the address of the user.
Identificator master	ID master	Numerical code that identifies the “master” data receiver module. This code has the physical address where it was located.
Day	DD	The day the “slave” data transmitter module sends a confirmation to the “master” data receiver module.
Month	MO	The month the “slave” data transmitter module sends a confirmation to the “master” data receiver module.
Year	YY	The year the “slave” data transmitter module sends a confirmation to the “master” data receiver module.
Hour	HH	The hour the “slave” data transmitter module sends a confirmation to the “master” data receiver module.
Minute	MM	The minute the “slave” data transmitter module sends a confirmation to the “master” data receiver module.

Detailed description of fields.

**Table 4:** Monitoring data frame. (**A**) Supervisor. Found people; (**B**) Supervisor. Missing people.

Start	Master ID	Slave ID	DD	MO	YY	HH	MM	DF	End
(**A**)									
@	1	A	29	06	16	22	19	!	&
@	1	A	29	06	16	22	20	!	&
@	1	A	29	06	16	22	21	!	&
@	1	A	29	06	16	22	22	!	&
@	1	A	29	06	16	22	23	!	&
@	1	A	29	06	16	22	24	!	&
@	1	A	29	06	16	22	25	!	&
@	1	A	29	06	16	22	26	!	&
(**B**)									
@		A						!	&

Data collected by the data transmitter breadboard. **Start**: “@” starts recording information. **Master ID**: Device identification “1, 2”. **Slave ID**: Device identification “A, B”. **DD**: Recording day. **MO**: Recording month. **YY**: Recording year. **HH**: Recording hour. **MM**: Recording minutes. **DF**: “!” dummy field. **End**: “&” end of the recording.

## Supplementary Materials

The following are available online at www.mdpi.com/2072-666X/8/10/292/s1.
