# Supplementary material for: Real Time Monitoring of Children, and Adults with Mental Disabilities Using a Low-Cost Non-Invasive Electronic Device
Source: Micromachines (Basel). 2017 Sep 28;8(10):292. doi: 10.3390/mi8100292 (PMC6190192; doi:10.3390/mi8100292)
Supplement: Supplementary file 1 [file micromachines-08-00292-s001.pdf]

Arduino source codes

```
/*
   Source code: Slave
   Project: Localization
   Carlos Polanco
   Ignacio Islas
   Dec, 29, 2016   */


#include <OneWire.h>
//#include <DallasTemperature.h>
//#include <Adafruit_GPS.h> // install the Adafruit GPS library
#include <SoftwareSerial.h> // load the Software Serial library
SoftwareSerial mySerial(5,4); // initialize the Software Serial ports 4 and 5
//Adafruit_GPS GPS(&mySerial); //create the GPS object

String inString = "";
String st_tem   = "";    // string to hold input
//String NMEA1; // variable for first NMEA sentence
//String NMEA2; // variable for second NMEA sentence


//int int_temperatura;   // variable to hold temperature
//int tem_int;
int v_ascci;

boolean stringComplete = false;  // whether the string is complete

//float temperatura = 0.0;
//float calculo = 0;

String inputString = "";         // a string to hold incoming data

char inicio ='@';   // start string
char fin ='&'; // end string
char identificador ='A'; // number of slave
char otro  ='!'; // optional field
//char dec_t;
char c; //to read characters coming from the GPS


#define ONE_WIRE_BUS 2     // data wire is plugged into port 2 on the Arduino
OneWire oneWire(ONE_WIRE_BUS);    // setup a oneWire instance to communicate with any
OneWire devices (not just Maxim/Dallas temperature ICs)
//DallasTemperature sensors(&oneWire);    // pass our oneWire reference to Dallas Temperature
```

```
void setup() {
    Serial.begin(9600); // open serial communications and wait for port to open:
  //  sensors.begin(); // start up the library

    Serial.begin(9600); // turn on serial monitor
/*     GPS.begin (9600); // turn on the GPS at 9600 bauds
    GPS.sendCommand("$PGCMD,33,0*6D"); // turn off antenna update nuisance data
    GPS.sendCommand("PMTK_SET_NMEA_UPDATE_10HZ"); // set update rate to 10 hz
    GPS.sendCommand("PMTK_SET_NMEA_OUTPUT_RMCGGA"); // request RMC and GGA
sentences only
    delay (1000); */
}

void loop() {

  /* readGPS();
   delay(12); */

   //sensors.requestTemperatures(); // Send the command to get temperature
   if (stringComplete) {
     inputString = "";   // clear the string
    /* temperatura = sensors.getTempCByIndex(0);  // get the temperature from module */

     st_tem+=inicio;    // to put the start on the string
     st_tem += identificador; // to put id slave on the string

    /* int_temperatura = temperatura * 100;
     tem_int = int_temperatura / 1000;
     v_ascci = (char)tem_int;
     st_tem+= v_ascci;

     tem_int = int_temperatura - (tem_int * 1000);
     dec_t = tem_int / 100;
     v_ascci = (char)dec_t;
     st_tem+= v_ascci;

     tem_int = tem_int - (dec_t * 100);
     dec_t = tem_int / 10;
     v_ascci = (char)dec_t;
     st_tem+= v_ascci;

     tem_int = tem_int - (dec_t * 10);
     v_ascci = (char)tem_int;
     st_tem+= v_ascci; */
```

```
      //Serial.print(st_tem);

  /*    Serial.print(GPS.longitude,4);
      Serial.print(GPS.latitude,4);
      Serial.print(GPS.altitude);

      if (GPS.day < 9) Serial.print('0');
      Serial.print(GPS.day, DEC);

      if (GPS.month < 9) Serial.print('0');
      Serial.print(GPS.month, DEC);

      Serial.print(GPS.year, DEC);


      if (GPS.hour < 9) Serial.print('0');
      Serial.print(GPS.hour, DEC);

      if (GPS.minute < 9) Serial.print('0');
      Serial.print(GPS.minute, DEC); */

      //Serial.print(otro);
      //Serial.print(fin);
    st_tem+= otro;
     st_tem+= fin;   // put end on the string
    Serial.print(st_tem);
      st_tem = "";   // clear the string



      stringComplete = false;
    }
}

void serialEvent() {
    while (Serial.available()) {
         char inChar = (char)Serial.read(); // get the new byte
         if (inChar == 'A') stringComplete = true;  // if the incoming character is a newline, set a flag so
the main loop can do something about it:
    }
}



/*void readGPS() {
    clearGPS();
    while (!GPS.newNMEAreceived()) {  // loop until you have a good NMEA sentence
```

```
        c = GPS.read ();
    }

    GPS.parse(GPS.lastNMEA());  // parse that last goo NMEA sentence
    NMEA1= GPS.lastNMEA();

    while (!GPS.newNMEAreceived()) {  // loop until you have a good NMEA sentence
        c = GPS.read ();
    }
    GPS.parse(GPS.lastNMEA());  // parse that last goo NMEA sentence
    NMEA2= GPS.lastNMEA();

// Serial.println(NMEA1);
// Serial.println(NMEA2);
// Serial.println("");

// Serial.println(GPS.latitude,4);
// Serial.println(GPS.lat);
// Serial.println(GPS.longitude,4);
// Serial.println(GPS.lon);
// Serial.println(GPS.altitude);


 // Serial.print("\nTime: ");
 // Serial.print(GPS.hour, DEC); Serial.print(':');
//  Serial.print(GPS.minute, DEC); Serial.print(':');
 // Serial.print(GPS.seconds, DEC); Serial.print('.');
 // Serial.println(GPS.milliseconds);
 // Serial.print("Date: ");
//  Serial.print(GPS.day, DEC); Serial.print('/');
 // Serial.print(GPS.month, DEC); Serial.print("/20");
 // Serial.println(GPS.year, DEC);
 // Serial.print("Fix: "); Serial.print((int)GPS.fix);
 // Serial.print(" quality: "); Serial.println((int)GPS.fixquality);
//  if (GPS.fix) {
//    Serial.print("Location: ");
 //   Serial.print(GPS.latitude, 4); Serial.print(GPS.lat);
  //  Serial.print(", ");
  //  Serial.print(GPS.longitude, 4); Serial.println(GPS.lon);
 //
  //  Serial.print("Speed (knots): "); Serial.println(GPS.speed);
   // Serial.print("Angle: "); Serial.println(GPS.angle);
  //  Serial.print("Altitude: "); Serial.println(GPS.altitude);
  //  Serial.print("Satellites: "); Serial.println((int)GPS.satellites);
 // }
```

```
} */


/*void clearGPS() {
    while (!GPS.newNMEAreceived()) {  // clear old and corrupt data from serial port
        c = GPS.read ();
    }
    GPS.parse(GPS.lastNMEA());  // parse that last goo NMEA sentence
    while (!GPS.newNMEAreceived()) {  // clear old and corrupt data from serial port
        c = GPS.read ();
    }
    GPS.parse(GPS.lastNMEA());  // parse that last goo NMEA sentence
    while (!GPS.newNMEAreceived()) {  // clear old and corrupt data from serial port
        c = GPS.read ();
    }
    GPS.parse(GPS.lastNMEA());  // parse that last goo NMEA sentence
}*/
```

```
/*
  Source code: Supervisor
  Project: Localization
  Carlos Polanco
  Ignacio Islas
  June, 16, 2017   */


#include <SoftwareSerial.h>



SoftwareSerial mySerial(10, 11); // RX, TX   for VDIP Module



#include <Wire.h>

#include "RTClib.h"

RTC_DS1307 RTC;



char masterID ='1';

char commingID = ' ';

String inputString = "";         // a string to hold incoming data

boolean VDIPReady = false;  // whether the string is complete

int cdorCara = 0;
```

```
char charAnt = '?';

int statusVDIP = 0;   //

int  ii, indexInStr = 0, indexIDSlaveTx = 0;

String noCaraFound_st = "";

int cero  = 0;

boolean flgStComplete = false, flgComMes = false, flgExp = false, flgIni = true;

String st_tem = "";

String slavesIDs[6] = "";

long contaRet = 320000;

unsigned int noCaraFound_int = 0, noEncontrados = 0, noEncontradosEnviados = 0, res_int = 0;

word pdw = 0;


void setup() {

  // initialize serial:

  Serial.begin(9600);   // rf tranceiver

    Wire.begin();

    RTC.begin();

  // reserve 200 bytes for the inputString:

  inputString.reserve(200);

   Serial.println("start up");


  // set the data rate for the SoftwareSerial port

  mySerial.begin(9600);
```

```
//  mySerial.println("---------");

  delay (3000);

  mySerial.print("DIR\r");
  /*
  delay (3000);
  mySerial.print("CLF MISSING.TXT\r");
  delay (3000);
  inputString = "";
  mySerial.print("CLF FOUND.TXT\r");
 */

}

void loop() {

  if (flgExp){
    contaRet--;
    if(contaRet == 0){
      contaRet = 320000;
      Serial.print(slavesIDs[indexIDSlaveTx]);
      indexIDSlaveTx++;
      if (indexIDSlaveTx > 4) indexIDSlaveTx = 0;
```

```
  }

}  // if explora

if (VDIPReady){
  VDIPReady = false;
  switch (statusVDIP) {
    case 0:
      mySerial.print("DIR MISSING.TXT\r");
      inputString = "";
      statusVDIP = 1;
      break;
    case 1:
      if (inputString[12] > 0) {
        inputString = "";
        statusVDIP = 2;
        mySerial.print("RD MISSING.TXT\r"); // leer datos
      }
      break;
    case 2:
//    mySerial.print("ready");
      for(ii=0; ii<5; ii++){
        slavesIDs[ii] = inputString[ii];   // respaldo de ids de esclavos
```

```
      }
      /*
      Serial.print(inputString[0]); // leer datos
      Serial.print(inputString[1]); // leer datos
      Serial.print(inputString[2]); // leer datos
      Serial.print(inputString[3]); // leer datos
      Serial.print(inputString[4]); // leer datos
      */
      inputString = "";
      statusVDIP = 20;
      mySerial.print("CLF MISSING.TXT\r");
      flgExp = true;  //

    break;
    case 3:
      inputString = "";
      statusVDIP = 4;
      mySerial.print("DIR FOUND.TXT\r");
    break;
    case 4:
/*
      Serial.print(inputString[11]); //
      Serial.print(inputString[12]); //
      Serial.print(inputString[13]); //
```

```
      Serial.print(inputString[14]); //
  */

     noCaraFound_int = (unsigned int)inputString[12];
     noCaraFound_int = noCaraFound_int << 8;
     res_int = (unsigned int)inputString[11];
//    noCaraFound_int = noCaraFound_int + res_int;
     noEncontrados = noCaraFound_int / 22;
     noEncontradosEnviados = 0;

     pdw = (byte)inputString[12];
     pdw = pdw << 8;
     pdw = pdw + byte(inputString[11]);
     noEncontrados = pdw / 22;
     Serial.print("C:");
     Serial.print(pdw);

//    Serial.print(inputString[12]);
//    Serial.print(inputString[11]);
//    Serial.print(noCaraFound_int);
//    Serial.print(res_int);

     Serial.print("Registros:");
```

```
Serial.println(noEncontrados);



 mySerial.print("OPR FOUND.TXT\r"); // open file to read
 inputString = "";
  statusVDIP = 5;
break;
case 5:
      mySerial.print("RDF ");  // read the string in the USB file
       mySerial.write(cero);
       mySerial.write(cero);
       mySerial.write(cero);
       mySerial.write(22);
       mySerial.print('\r');
  inputString = "";
  statusVDIP = 6;
break;
case 6:
       for (ii=0; ii<22; ii++) Serial.print(inputString[ii]);
       noEncontradosEnviados ++;
       if (noEncontrados > noEncontradosEnviados){
         mySerial.print("RDF ");  // read the string in the USB file
         mySerial.write(cero);
         mySerial.write(cero);
```

```
        mySerial.write(cero);
        mySerial.write(22);
        mySerial.print('\r');
        inputString = "";
        statusVDIP = 6;
      }else {
        mySerial.print("CLF FOUND.TXT\r");
        inputString = "";
        statusVDIP = 20;    // without case
        flgExp = false;
        flgExp = true;
      }
  break;
//---------------------------
  case 7:
   mySerial.print("WRF ");  // write the string in the USB file
   mySerial.write(cero);
   mySerial.write(cero);
   mySerial.write(cero);
   mySerial.write(22);
   mySerial.print('\r');
   //mySerial.print(st_tem);
   for (ii=0; ii<20; ii++) mySerial.print(st_tem[ii]);
```

```
  inputString = "";
  statusVDIP = 8;
  mySerial.print("\r\n");

/*
  for (ii=0; ii<20; ii++) Serial.print(inputString[ii]);
  noEncontradosEnviados ++;
  mySerial.print("CLF FOUND.TXT\r");
  inputString = "";
  statusVDIP = 8;
*/

case 8:
  inputString = "";
  statusVDIP = 20;    // without case
  flgExp = true;
  mySerial.print("CLF FOUND.TXT\r"); // close the USB file
break;


default:



break;
```

```
  } // del switch
  }   //  if VDIPReady
  if (mySerial.available()) {
    char inChar = (char)mySerial.read();
  //  mySerial.print(inChar);
    // add it to the inputString:
    inputString += inChar;
    // if the incoming character is a newline, set a flag
    // so the main loop can do something about it:
    if ((inChar == '\r' ) && (charAnt == '>')) {
//     mySerial.print('s');
      VDIPReady = true;
    }
    charAnt = inChar;
  }

  /*
  if (Serial.available()) {
    mySerial.write(Serial.read());
  }
*/
```

```
/*
// print the string when a newline arrives:
if (VDIPReady) {
  mySerial.print(inputString);
  // clear the string:
  inputString = "";
  VDIPReady = false;
}
*/

if (flgStComplete) {

       if((commingID >= 'A') && (commingID <= 'Z')){   // answer of slave
         flgExp = false;
         Serial.print("llega esclavo");
         st_tem = "";
         st_tem +='@';
         st_tem +=commingID;
         st_tem +='!';
         st_tem +='&';

         DateTime now = RTC.now();
         st_tem += (now.year());
         st_tem +='/';
```

```
 if (now.month() >= 0 && now.month() < 10) {
    st_tem +='0';
 }
 st_tem +=(now.month());
 st_tem += '/';
 if (now.day() >= 0 && now.day() < 10) {
    st_tem +='0';
}
st_tem +=(now.day());
st_tem += masterID;
if (now.hour() >= 0 && now.hour() < 10) {
   st_tem +='0';
}
st_tem +=(now.hour());
st_tem += ':';
if (now.minute() >= 0 && now.minute() < 10) {
   st_tem +='0';
}
st_tem +=(now.minute());

Serial.print(st_tem);
/*-*/    mySerial.print("OPW FOUND.TXT\r"); // open to write file
 inputString = "";
```

```
      statusVDIP = 7;

    }
    else if((commingID >= '1') && (commingID <= '9')){   // asking supermaster
      flgExp = false;
      Serial.print("supermaestro");
      statusVDIP = 3;
      mySerial.print("DIR\r");
    }

    flgStComplete = false;
  }

}

/*
  SerialEvent occurs whenever a new data comes in the
 hardware serial RX.  This routine is run between each
 time loop() runs, so using delay inside loop can delay
 response.  Multiple bytes of data may be available.
```

```
 */


void serialEvent() {
  while (Serial.available()) {
    // get the new byte:
    char inChar_2 = (char)Serial.read();
//   Serial.print(inChar);
    // add it to the inputString:
    inputString += inChar_2;
    // if the incoming character is a newline, set a flag
    // so the main loop can do something about it:
    if (inChar_2 == '@' ){
      indexInStr = 0;
      flgComMes = true;
      inputString = "";
    }
    if (inChar_2 == '&' &&  flgComMes == true){
      commingID = inputString[0];
      flgComMes = false;
      flgStComplete = true;
    }
  }
```

```
}
```

```
/*
   Source code: Supervisor
   Project: Localization
   Carlos Polanco
   Ignacio Islas
```

```
  January, 24, 2017   */

#include <SoftwareSerial.h>
SoftwareSerial mySerial(10, 11); // RX, TX


/*#include <Wire.h>
#include "RTClib.h"
RTC_DS1307 RTC; */

const int buttonPin2 = 2;     // push to send request
const int buttonPin3 = 4;     // push to close the file
const int ledPin    = 13;      // the number of the LED pin

int flag   = 0;
int espera  = 1;
int flag_inicio = 0;

int cero  = 0;
int buttonState2  = 1;         // variable for reading the pushbutton2 status
int buttonState3  = 1;         // variable for reading the pushbutton3 status
int esclavomax    = 1;
int identificador = 1;
int j;
int k;
int longitud;
int long_esclavo;
int longitud_archivo;
int numero_registros;
int index = 1;
int registros_enviados =0;
int nueva = 22;

int day_alarm = 02; // Dia alarma

double conta = 170000;


char inicio ='@';   // start string
char fin ='&'; // end string
char identificadorA = 'A'; // number of slave
char identificadorB = 'B';
char identificadorC = 'C';
char identificadorX = 'X';

char otro  ='!'; // optional field
```

```
String st_tem = "";
String st_serial = "";
String lectura ="";
String st_extraviados ="";
String st_consulta ="@1&";




boolean stringComplete = false;

void setup() {

   Serial.begin(9600);
 /*  Wire.begin();
   RTC.begin();
 if (! RTC.isrunning()) {
   Serial.println("RTC is NOT running!");
   // following line sets the RTC to the date & time this sketch was compiled
   RTC.adjust(DateTime(__DATE__, __TIME__)); */
// }

    mySerial.begin(9600);
    pinMode(ledPin, OUTPUT); // initialize the LED pin as output
    pinMode(buttonPin2, INPUT); // initialize the pushbutton2 pin as input
    pinMode(buttonPin3, INPUT);  // initialize the pushbutton3 pin as input
}

void loop() {
       if (flag_inicio == 0) { //  file lenght
          mySerial.print("DIR MISSING.TXT\r"); // obtengo longitud
          delay (2000);
          flag_inicio = 1;
       }

   while (mySerial.available()) {
          char inChar = (char)mySerial.read(); // get the new byte
          st_serial += inChar; // add it to the st_tem
          if (inChar == '>')  {
            if (flag_inicio == 1) flag_inicio = 2;

            if (flag_inicio ==3) flag_inicio = 4;

             if (flag_inicio ==6) flag_inicio = 7;
```

```
        if (flag_inicio ==8) flag_inicio = 9;

        if (flag_inicio ==10) flag_inicio = 11;

      }  // if the incoming character is a newline, set a flag, so the main loop can do something about it
  }

  if (flag_inicio == 2)  {
    flag_inicio = 3;
   longitud = (int)st_serial[11];
   if (st_serial[12] > 0) {
      st_serial = "";
      mySerial.print("RD MISSING.TXT\r"); // leer datos
      delay (2000);

   }
 }
 if (flag_inicio == 4) {


  st_extraviados+=inicio;    // to put the start on the string
     st_extraviados += identificador; // to put id slave on the string
         for (j == 1; j< 5;j++){
    st_extraviados+=  (char)st_serial[j];
   }
         st_extraviados+= otro;
    st_extraviados+= fin;   // put end on the string
   Serial.print(st_extraviados);

   flag_inicio = 5;
 }

    if (flag_inicio == 7)  {
    flag_inicio = 8;

   longitud_archivo = (int)st_serial[11];
Serial.write(nueva);
Serial.write(longitud_archivo);
   Serial.write(st_serial[11]/nueva);
 //   Serial.print(st_serial[12]);
  //longitud_archivo = longitud_archivo/nueva;
   numero_registros = (int)(longitud_archivo/nueva);
//    numero_registros = 8;
    // nueva = 22;
   Serial.write(numero_registros);
//    Serial.print ("XXX");
//    Serial.print ((char)st_serial[8]);
```

```
  if (st_serial[11] > 0) {
    st_serial = "";
    mySerial.print("OPR FOUND.TXT\r"); // open file to read
    delay (2000);
    registros_enviados = 0;
  }
}

  if (flag_inicio == 9) {
     flag_inicio = 10;
     st_serial = "";
     mySerial.print("RDF ");  // read the string in the USB file
         delay (2000);
         mySerial.write(cero);
         mySerial.write(cero);
         mySerial.write(cero);
         mySerial.write(22);
         mySerial.print('\r');
         delay (2000);
   /*   mySerial.print("RDF ");  // read the string in the USB file
         delay (2000);
         mySerial.write(cero);
         mySerial.write(cero);
         mySerial.write(cero);
         mySerial.write(22);
         mySerial.print('\r');
         delay (2000); */
  }

  if (flag_inicio == 11) {
     //   for (k == 1; k<= 22;k++){
      //   Serial.print(st_serial[k]);
      //  }
       registros_enviados++;
       Serial.print(registros_enviados);

       if (registros_enviados < numero_registros){
         Serial.print("sigueenviando");
          flag_inicio = 10;
          st_serial = "";
          mySerial.print("RDF ");  // read the string in the USB file
          delay (2000);
          mySerial.write(cero);
          mySerial.write(cero);
          mySerial.write(cero);
          mySerial.write(22);
          mySerial.print('\r');
```

```
           delay (2000);
         } else {
           mySerial.print("CLF FOUND.TXT\r"); // close the USB file
           flag_inicio = 12;
           Serial.print("termine");
         }

    }

    buttonState2 = digitalRead(buttonPin2);
    buttonState3 = digitalRead(buttonPin3);



    if (stringComplete) {

         if (st_tem[1] >= 49 && st_tem[1] <= 57) { // recibe information del supervisor son numeros


           if (long_esclavo == 7) {

//             mySerial.print("DLF MISSING.TXT\r"); // delete the MISSING.TXT file
  //           delay (2000);
           mySerial.print("OPW MISSING.TXT\r");
           mySerial.print("WRF ");  // write the string in the USB file
           delay (2000);


           mySerial.write(cero);
           mySerial.write(cero);
           mySerial.write(cero);
           mySerial.write(5);
           mySerial.print('\r');
           delay (2000);
           mySerial.print(st_tem[2]);
           mySerial.print(st_tem[3]);
           mySerial.print(st_tem[4]);
           mySerial.print(st_tem[5]);
           mySerial.print(st_tem[6]);
         //  mySerial.print("\r\n");
           delay (2000);
          mySerial.print("CLF MISSING.TXT\r"); // close the USB file
           delay (2000);
           st_tem = "";
           stringComplete = false;
           }
           if (long_esclavo == 2){
```

```
          st_serial = "";
          mySerial.print("DIR FOUND.TXT\r"); // obtengo longitud
          flag_inicio = 6;
          long_esclavo = 0;
        }

    } else {  // recibe informacion del esclavo

/*    DateTime now = RTC.now();

    st_tem += (now.year());
    st_tem +='/';

    if (now.month() >= 0 && now.month() < 10) {
      st_tem +='0';
    }
    st_tem +=(now.month());
    st_tem += '/';

    if (now.day() >= 0 && now.day() < 10) {
      st_tem +='0';
    }
   st_tem +=(now.day());
   st_tem += '1';

   if (now.hour() >= 0 && now.hour() < 10) {
     st_tem +='0';
   }
   st_tem +=(now.hour());
   st_tem += ':';

   if (now.minute() >= 0 && now.minute() < 10) {
     st_tem +='0';
   }
   st_tem +=(now.minute()); */


  //if (flag == 0) { // open the USB file
   // flag = 1;
  mySerial.print("OPW FOUND.TXT\r"); // open to write file
  delay (2000);
 // }
  mySerial.print("WRF ");  // write the string in the USB file
  //delay (2000);
  mySerial.write(cero);
  mySerial.write(cero);
  mySerial.write(cero);
```

```
    mySerial.write(22);
    mySerial.print('\r');
    delay (2000);
    mySerial.print(st_tem);
    mySerial.print("\r\n");
    delay (2000);
    st_tem = "";
    conta = 0;
    delay(5000);
    mySerial.print("CLF FOUND.TXT\r"); // close the USB file
    delay (2000);
    stringComplete = false;
  }

  }

  if (conta > 0)  conta--;

/*     if (buttonState3 == LOW) {
      digitalWrite(ledPin, HIGH);
      delay(2000);
    //  mySerial.print("CLF FOUND.TXT\r"); // close the USB file
      delay (2000);
      digitalWrite(ledPin, LOW);
      index = 9;

    } */



  if (conta == 0) {

    if (index == 1) {
      st_consulta[1] ='1';
      Serial.print(st_consulta);
      conta =  170000;
      index = 2;
    }
    else if (index == 2) {
      st_consulta[1] ='2';
      Serial.print(st_consulta);
      conta =  170000;
      index = 3;
    }
    else if (index == 3) {
      st_consulta[1] ='3';
      Serial.print(st_consulta);
```

```
        conta =  170000;
        index = 1;
      }
    }
}


void serialEvent() {
    while (Serial.available()) {
        char inChar = (char)Serial.read(); // get the new byte
        st_tem += inChar; // add it to the st_tem

        if (inChar == '@') {
          long_esclavo = 0;
        }
        else long_esclavo++;

        if (inChar == '&')  stringComplete = true;  // if the incoming character is a newline, set a flag, so
the main loop can do something about it
    }
}
```
